# A wide dynamic range diamond quantum sensor as an electric vehicle battery monitor

**DOI:** 10.1098/rsta.2022.0312

**Published:** 2024-01-22

**Authors:** Yuji Hatano, Junya Tanigawa, Akimichi Nakazono, Takeharu Sekiguchi, Shinobu Onoda, Takeshi Ohshima, Takayuki Iwasaki, Mutsuko Hatano

**Affiliations:** ^1^ Department of Electrical and Electronic Engineering, School of Engineering, Tokyo Institute of Technology, Meguro-ku, Tokyo, Japan; ^2^ Yazaki Research and Technology Center, YAZAKI Corporation, Susono, Shizuoka, Japan; ^3^ Quantum Materials and Applications Research Center (QUARC), National Institutes for Quantum Science and Technology (QST), Takasaki, Gunma, Japan

**Keywords:** diamond quantum sensor, nitrogen-vacancy centre, current sensor, multi-modal sensor, magnetic field sensor, electric vehicle battery monitor

## Abstract

To demonstrate the application capability of the diamond quantum sensor as an electric vehicle (EV) battery monitor, we (i) investigated the measurable current in a real car noise level and (ii) compared the linearity with conventional sensors. Consequently, (i) we could measure a 20 mA current pulse even under an external magnetic field of 80 µT, which is larger than that of 50 µT around the EV battery module in a real car during driving. The 20 mA pulse measurement corresponds to the EV battery state of charge estimation accuracy of 0.2% in the standard driving pattern, which is smaller than the present level of 10%. (ii) The linearity degradation seen in the Hall sensor near the upper limit of the measurement range was not seen in the diamond sensor. Although the Hall sensor and the shunt resistor showed linearity degradation in the current range of several tens of amperes or less, the degradation was smaller for the diamond sensor. The transverse magnetic field effect in the diamond sensor on the linearity was estimated to be less than 0.01% for a several-degree misalignment of the sensor surface to the magnetic field direction and under a 340 A current.

This article is part of the Theo Murphy meeting issue 'Diamond for quantum applications'.

## 1. Introduction

**Figure 1.**
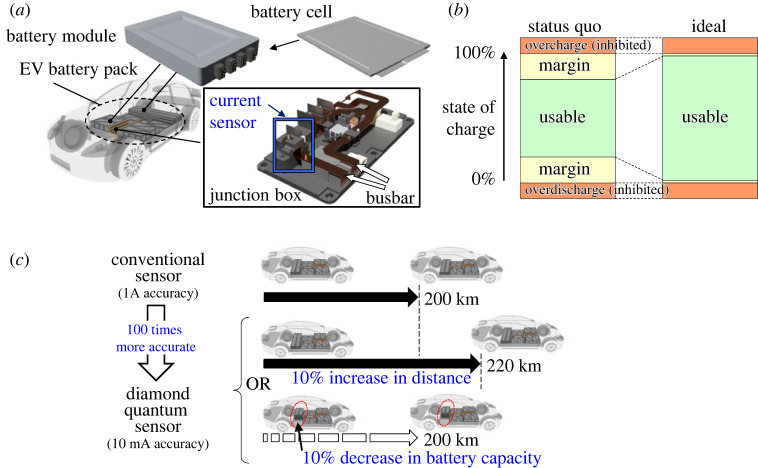
Electric vehicle (EV) battery monitor. (*a*) Connection to measure the current in the busbar from the battery module. (*b*) Usable state of charge (SOC) range of the battery. (*c*) Expected effect of the battery monitor with enhanced accuracy.

Owing to the recent growth in the electrification of automobiles, the requirement for a highly accurate current sensor for the state of charge (SOC) estimation of electric vehicle (EV) is increasing. The EV battery monitor measures the current in the busbar from the battery pack, as shown in figure 1*a*. The EV Battery pack has a large capacity by consisting of multiple battery modules. *A high-performance current sensor is required* to minimize the battery capacity, use the storage capacity without waste, extend the lifetime of the battery and realize safe usage. The requirement for battery monitoring with high accuracies results from the relatively large margin in the status quo SOC compared with the usable range, as shown in figure 1*b*. Battery capacity usage is enabled without waste by compressing the margin by improving the accuracy of the battery monitor. Presently, a typical EV battery monitor has an accuracy of approximately 1 A because the maximum value of the charging and discharging currents exceed ±100 A. However, the average is approximately 10 A. The average value of an EV with the standard weight in the worldwide light-vehicle test cycle (WLTC) current pattern is 14 A [1]. In constant-speed driving, the current even decreases. Suppose a sensor with an accuracy of 1A is used to calculate the cumulative value of a current of approximately 10 A. In that case, a margin of 10% should be required. Suppose the accuracy of the sensor is 10 mA while keeping the same measurable current range. In that case, the margin can be reduced to 0.1%. Thus enhancing the accuracy of the battery monitor from 1 A to 10 mA while maintaining the maximum measurable current range extends the cruising mileage by 10% with the same battery or reduces the battery volume by 10% with the same cruising mileage, as shown in figure 1*c*.

Diamond quantum sensors including NV-centre are characterized by high sensitivities [2–22] and wide dynamic ranges [23–25], and are suitable for automotive applications. Thus, we investigated a diamond quantum sensor for EV battery monitoring and have realized an accuracy of 10 mA and dynamic range of ±1000 A [1]. However, to promote the diamond quantum sensor to real applications such as vehicular technology, further evaluation of the application capability was required for the following issues.

(i) Measurable current in a real car noise level.
(ii) Linearity comparison with conventional sensors.

**Figure 2.**
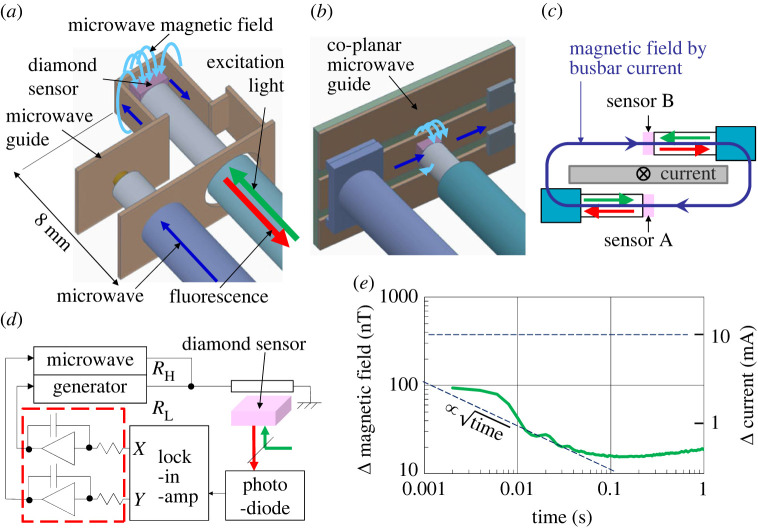
EV battery monitor to measure the busbar current with the diamond sensor. (*a*) The sensor head comprising a diamond sensor and the surrounding microwave guide. The microwave magnetic field generated by the microwave current is shown as arc arrows, which is perpendicular to the [111] NV-axis of the diamond sensor. (*b*) The previously used sensor head [20] comprising a co-planar type microwave guide, which also generated the microwave magnetic field perpendicular to the [111] NV-axis but required a larger distance from the busbar than the one in (*a*) to maintain the (111) surface of the diamond sensor perpendicular to the busbar surface. (*c*) Dual sensor heads placed on both sides of the busbar for differential measurement to eliminate external magnetic field noise as a common mode. (*d*) Integration feedback control circuits surrounded by dashed box to measure the busbar current as the relative RFD, ΔRFD ≡ Δ(*R*_H_ − *R*_L_) and to measure the SOC as its accumulation, where *R*_H_ and *R*_L_ are the higher and lower microwave resonance frequencies, respectively. (*e*) Fluctuation of the measured magnetic field in the laboratory during the day without magnetic shielding as a function of the measurement time, obtained as the Allan deviation of (RFD_B – RFD_A)/2*γ*, where RFD_A and RFD_B are RFDs of sensors A and B, respectively. It decreases proportional to the square root of the measurement time below 50 ms with some bouncing possibly due to 50 Hz noise and its harmonics. Slight increase above 100 ms is reflecting low frequency environmental noise. The right axis shows the current scale obtained using the measured relation 37 nT mA^−1^. The measurable current was below 10 mA above 0.002 s.

**Figure 3.**
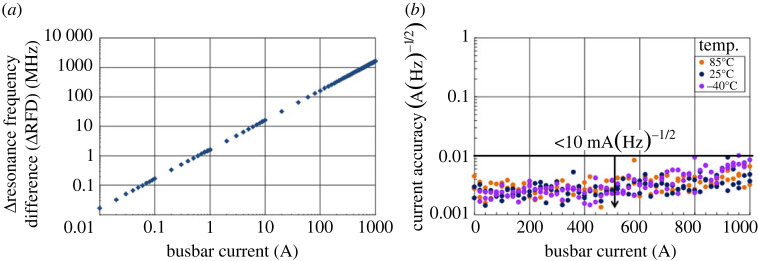
Busbar current dependence of (*a*) amplitude and (*b*) noise level of the sensor output as the relative resonance frequency difference.

**Figure 4.**
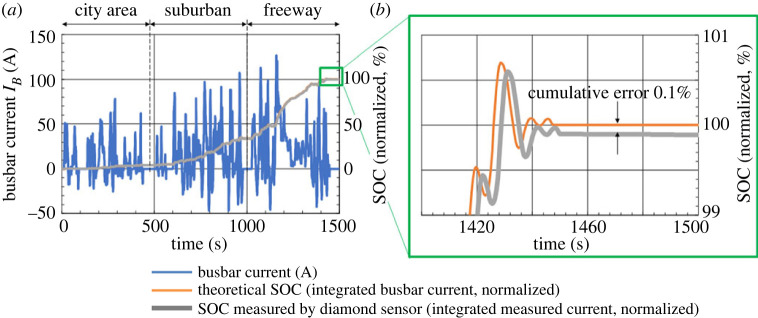
SOC measured for the worldwide light-vehicle test cycle (WLTC) current pattern. (*a*) The WLTC current pattern comprising city area, suburban and freeway portion, obtained by assuming a standard automobile weight. The theoretical and measured SOC, which are the integrated busbar and measured current, normalized to the final theoretical SOC value as 100%, overlapped. (*b*) Theoretical and measured SOC, expanded in the last 100 s of the WLTC pattern.

This study aims to introduce our initial experiment for the aforementioned issues. First, we overviewed the diamond quantum sensor as an EV battery monitor with an accuracy and dynamic range of 10 mA and ±1000 A, respectively, as the background. Figures 1–4 used in this overview were adapted from [1]. Subsequently, we described the set-ups and experimental results for (i) and (ii) as further evaluation of the application capability. Furthermore, we estimated the transverse magnetic field effect observed in the experiment of (ii) on the linearity. Eventually, we summarized the results obtained for (i) and (ii) and the remaining future tasks.

## 2. Overview of the diamond quantum sensor as an electric vehicle battery monitor

This section outlines the diamond quantum sensor for EV battery monitoring with 10 mA accuracy and ±1000 A dynamic range to improve the battery usage efficiency by 10%. As the operating principle of the NV-centre included in the diamond quantum sensor, a longitudinal magnetic field *B*_‖_ parallel to the NV-axis causes Zeeman splitting between the lower and higher microwave resonance frequencies *R*_L_ and *R*_H_, which are observed as dips in the fluorescence spectrum. The difference between the two resonance frequencies proportional to the longitudinal magnetic field, *R*_H_ − *R*_L_ = 2*γB*_‖_ (*γ*: the gyromagnetic ratio), is referred to as the resonance frequency difference (RFD) hereinafter.

The EV battery monitor measures the busbar current from the battery module with the diamond sensor as shown in figure 2. The sensor head shown in figure 2*a* is the diamond sensor adhered to the fibre top and surrounded by the microwave guide within a dimension of 5 × 10 × 10 mm^3^. The diamond sensor is a type Ib (111) crystal synthesized by a high-pressure high-temperature process from Sumitomo Electric with a dimension of 2 × 2 × 1 mm^3^. A 3 × 10^18^ cm^−2^, 2 MeV electron beam irradiation and the subsequent annealing at 1000°C for 2 h generated 5–6 ppm nitrogen-vacancy (NV) centre from approximately 100 ppm of nitrogen. The microwave guide provided a magnetic field perpendicular to the [111] NV-axis.

The previously used co-planar microwave guide [20], which also generated the microwave magnetic field perpendicular to the [111] NV-axis is shown in figure 2*b*. The central conductor of the co-planar microwave guide was bent along the diamond sensor surface to form the microwave guide shown in figure 2*a* to shorten the distance from the diamond sensor to the busbar and enhance the sensitivity to the busbar current.

Two sensors, A and B were placed below and above the busbar for differential measurement [26–29], as shown in figure 2*c*. The external noise was eliminated as a common mode extracting the difference between the two sensor outputs. A shorter distance from the diamond sensor to the busbar was also effective to enhance the common mode noise rejection ratio.

The control system used to trace the RFD is shown in figure 2*d*. This integration feedback control from the lock-in-amplifier to the microwave generators enabled the measurement of the busbar current as the relative RFD and also cancelled the difference between the accumulated current, which equals SOC, and the accumulated relative RFD, thus realizing exact equality between them.

The fluctuation of the measured magnetic field in the laboratory during the day without magnetic shielding as a function of the measurement time, obtained as the Allan deviation of (RFD_B – RFD_A)/2*γ*, where RFD_A and RFD_B are RFDs of the sensors A and B respectively, is shown in figure 2*e*. The fluctuation decreases almost proportional to the square root of the measurement time below 50 ms, although some bouncing possibly due to 50 Hz noise and its harmonics was overlapped. It also slightly increases above 100 ms reflecting low frequency environmental noise. The right axis shows the current scale obtained using the measured relation 37 nT mA^−1^. From this figure, we could confirm that the measurable current was below 10 mA above 0.002 s.

Using the fibre-top diamond sensor, differential configuration and feedback control system shown in figure 2, a wide dynamic range of currents up to ±1000 A was observed, as shown in figure 3*a*. Moreover, current accuracies of better than 10 mA (Hz)^−1/2^ were validated in the temperature range of −40 to 85°C, the range is the performance requirement for an EV, as shown in figure 3*b*. Measurement was conducted in an unshielded environment of our laboratory. These outstanding performances are the superior features of diamond quantum sensors.

Furthermore, as the standard driving mode, the WLTC current pattern, consisting of the city area, suburban and freeway portions, as shown in figure 4*a*, was measured. The busbar current was obtained from the WLTC driving pattern by assuming a typical automobile weight and motor engine performance. In the same figure, the SOC measured by the diamond sensor and the theoretical SOC, which is the integration of the busbar current, overlapped. The measured and theoretical SOC were normalized to the final theoretical SOC value as 100%. They coincided within a cumulative error of 0.1%, as shown in figure 4*b*, validating the tracking capability and accuracy of the diamond sensor.

Based on these results, assuming this sensor is implemented for all global EV and plug-in-hybrid EV (PHEV) sales in 2030 (20 million units), the emission of CO_2_ will be reduced by 14 million tons. This corresponds to 0.2% of the CO_2_ emitted by the global transportation activities and industries (8 billion tons) or the CO_2_ emitted by two thermal power plants with capacities of 1 million kWh each. This also saves lithium, a resource with limited reserves.

## 3. Further evaluation of the application capability

Next, we conducted experiments to validate the following issues to validate the application capability.

(i) Measurable current in a real car noise level.
(ii) Linearity comparison with conventional sensors.

**Figure 5.**
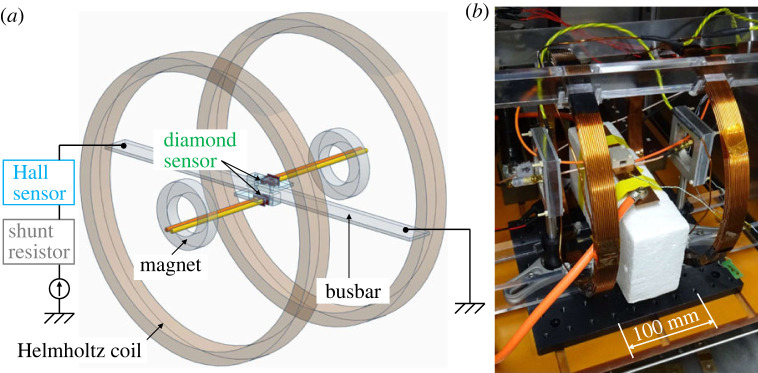
(*a*) Illustration and (*b*) photograph of the static magnetic field set-up surrounding the busbar and the diamond sensor as well as the serial connection of conventional sensors to the busbar used to evaluate the quantitative effect of the external magnetic field noise on the measurability of the diamond sensor and the linearity comparison of the diamond sensor with conventional sensors for a large current amplitude.

The static magnetic field set-up surrounding the busbar and the diamond sensor as well as the serial connection of conventional sensors to the busbar for experiments (i) and (ii) are shown in figure 5. For experiment (i), Helmholtz coils to generate an external magnetic field to reproduce a real car noise level were placed on both sides of the busbar in addition to the magnets for static magnetic fields. For experiment (ii), the Hall sensor and shunt resistor generally used in present EVs were connected to the busbar in series to measure the same current as the diamond sensor.

**Figure 6.**
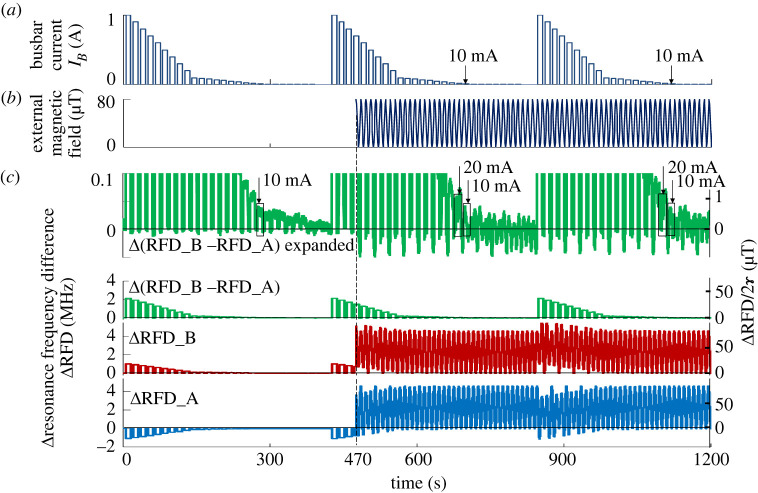
Test results of the external magnetic field noise effect on the measurability of the diamond sensor. (*a*) Current pulse train from 1 A to 1 mA. (*b*) 80 μT external magnetic field of a 0.1 Hz sine wave starting from 470 s. (*c*) Outputs of diamond sensors A and B as well as their difference as the relative resonance frequency difference.

The Helmholtz coil had an inner diameter of 230 mm and an outer diameter of 250 mm. An external magnetic field of 0–80 µT was generated in the diamond sensors placed below and over the busbar by providing a sinusoidal waveform of 0–150 mA with 75 mA offset to the coil at 0.1 Hz. For experiment (i), a pulse train from 1000 to 1 mA was applied to the busbar (with a time interval of 10 s ON and 5 s OFF), as shown in figure 6*a*. The external magnetic field was initially zero and was supplied after 470 s, as shown in figure 6*b*. The external magnetic field around the EV battery module during driving had been evaluated to reach 50 µT, less than 80 µT generated by the coil.

The magnetic fields measured by diamond sensors A and B as the relative resonance frequency difference (ΔRFD) are shown in figure 6*c* as ΔRFD_A and ΔRFD_B, and their difference as Δ(RFD_B − RFD_A), respectively. Neither ΔRFD_A nor ΔRFD_B could measure even a 1 A pulse in the presence of an external magnetic field of 80 µT. However, as Δ(RFD_B − RFD_A) in figure 6*c* increased, a 20 mA pulse could be discriminated with a 10 mA pulse, even under an external magnetic field noise of 80 µT. As previously mentioned, the measurable current was 10 mA and the SOC estimation accuracy was 0.1% in an unshielded environment but without intentional external noise. The SOC estimation accuracy degradation is predicted to be approximately 0.2% even if the detection current becomes 20 mA owing to in-vehicle noise. The improvement from the current estimation accuracy of 10–0.1% or 0.2% makes no significant difference and has no impact on CO_2_ reduction. To further improve measurability from 20 mA, the distance between the two differential sensors should be decreased to increase the common-mode rejection ratio.

**Figure 7.**
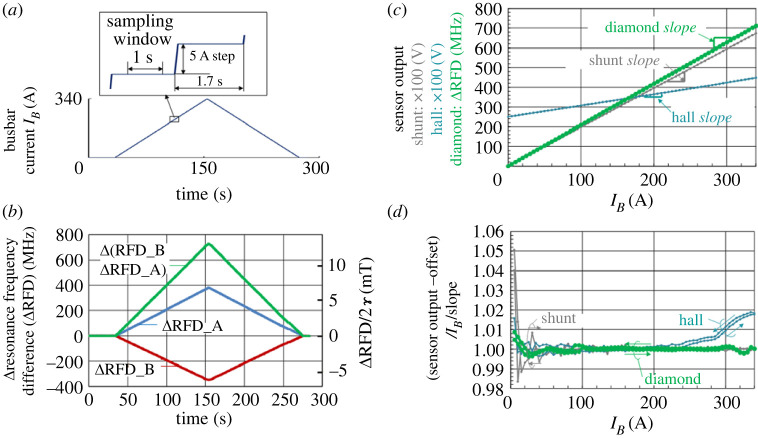
Linearity test results for a large current amplitude of the diamond sensor compared with conventional sensors. (*a*) Triangular-like current pattern with an amplitude of 340 A provided in steps of 5 A. (*b*) Outputs of diamond sensors A and B as well as their difference as the relative resonance frequency difference. (*c*) Comparison of the outputs of the Hall sensor, the shunt resistor and the diamond sensor versus busbar current. The vertical axis of the diamond sensor is Δ(RFD_B − RFD_A) in MHz, 100 times the output voltage for the Hall sensor and 100 times the output voltage for the shunt resistor. (*d*) Linearity comparison of the Hall sensor, the shunt resistor and the diamond sensor versus busbar current, defined as the normalized gain, which is the difference between the sensor output and the offset divided by the busbar current and the slope.

A Hall element sensor with a measurable range of 350 A and a shunt resistor with a range of 500 A were used for experiment (ii). According to these measurable ranges, a busbar current with a peak value of 340 A was applied in a triangular-like waveform from 0 A → 340 A → 0 A in steps of 5 A, as shown in figure 7*a*. As the output of the diamond sensor, either ΔRFD_A or ΔRFD_B could reproduce a waveform similar to that of the provided input and Δ(RFD_B − RFD_A) could reproduce the summed amplitude waveform, as shown in figure 7*b*.

Subsequently, the average value of each sensor output obtained within a flat sampling window in each step was plotted against the busbar current, as shown in figure 7*c*. All the sensors showed almost linear lines at first glance. The normalized gain defined below was obtained to investigate the linearity in detail, as shown in figure 7*d*.

— *X_i_* and *Y_i_* are the *i*th busbar current and sensor output, respectively.
— *a* and *b* are the least-squares fits of *Y_i_* = *a* × *X_i_* + *b*.
— The normalized gain defined as (*Y_i_* −*b*)/*a*/*X_i_* versus *X_i_* was plotted for each sensor.

In discussing figure 7*d*, the accuracy of the current source (Kikusui PAT20-400) is 0.5%. In addition, only one sensor was used for each measurement, and variations among individual sensors may have influenced the results. However, the following qualitative trends were observed.

— The Hall sensor had a larger linearity error compared with the diamond sensor near the upper end of their range, probably owing to saturation.
— The shunt resistor and the Hall sensor both have larger linearity errors in the low current range (several tens of amperes or less) compared with the diamond sensor, probably owing to the smaller voltage output.

For a detailed discussion of linearity, improvement of the accuracy of the current source and the evaluation of multiple sensors of the same type would be necessary.

In summary,

(i) A 20 mA current pulse could be measured even under an external magnetic field noise of 80 µT, which is larger than that of 50 µT around the EV battery module during driving in a real car.
(ii) The linearity degradation seen in the Hall sensor near the upper limit of the measurement range was not seen in the diamond sensor. Although the Hall sensor and the shunt resistor measurement errors increased in the small current range of several tens of amperes or less, the increase was smaller for the diamond sensor.

## 4. Estimation of the transverse magnetic field effect on the linearity

**Figure 8.**
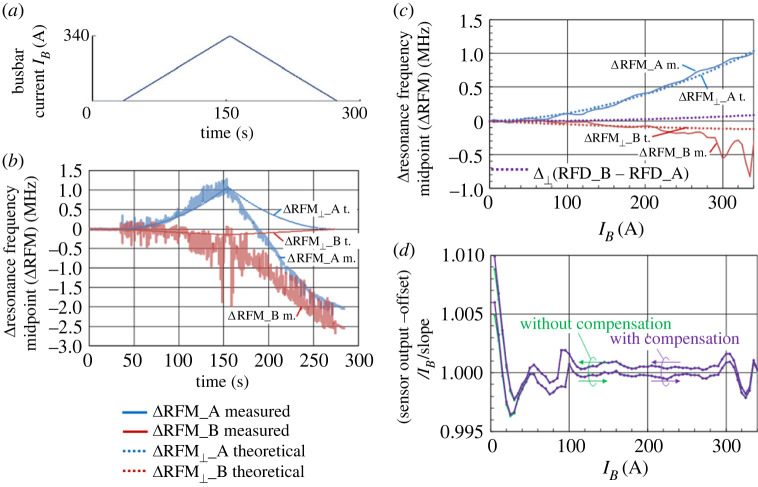
Transverse magnetic field effect on the relative resonance frequency midpoint and linearity. (*a*) Triangular-like current pattern with an amplitude of 340 A provided with steps of 5 A. (*b*) Measured relative resonance frequency midpoints (ΔRFM) of diamond sensors A and B as well as their theoretical values considering the transverse magnetic field effect (ΔRFM_⊥_). (*c*) Measured and theoretical relative resonance frequency midpoints (ΔRFM, ΔRFM_⊥_) in the rising steps versus current. The theoretical transverse magnetic field effect on the relative resonance frequency difference is shown in dotted line as Δ_⊥_(RFD_B − RFD_A). The legend of ΔRFM and ΔRFM_⊥_ is common to (*b*) and (*c*). (*d*) Normalized gain of the relative resonance frequency difference Δ(RFD_B − RFD_A) of the diamond sensor with and without the compensation of the transverse magnetic field effect. There was no meaningful difference between them.

In experiment (ii), some phenomena that seemed to be caused by the transverse magnetic field were observed. We focused on the midpoint of the resonance frequency RFM ≡ (*R*_H_ + *R*_L_)/2 when an input current of 340 A was applied, as shown in figure 8*a*. ΔRFM was expected to reflect the temperature-dependent zero-field splitting at approximately 74 kHz °C^−1^ at approximately room temperature [30–33]. However, as shown in figure 8*a*, the observed ΔRFM was also related to the applied current. In sensor A below the busbar, which was supposed to receive less heating effect of the busbar than sensor B above the busbar, ΔRFM_A showed a larger change compared with ΔRFM_B and the sign was contrary to the temperature increase.

For such changes in ΔRFM_A and ΔRFM_B, we suspected the effect of the transverse magnetic field caused by the misalignment between the [111] NV-axis and external magnetic field. The external magnetic field consists of the static magnetic field provided by the magnet and the busbar current magnetic field. We defined two angles: *θ*, the angle between the [111] axis and the static magnetic field *B*_0_; *φ*, the angle between the [111] axis and busbar current magnetic field *αI* (*α* is the proportionality constant and *I* is the current).

The energy levels of the *m* = ±1 and 0 states of the spin triplet of the NV-centre are approximated to the second-order perturbation of the transverse field.

$$E_{\pm 1}=f_{c}\pm \omega_{B∥}+\frac{\omega_{B⊥}^{2}/2}{f_{c}\pm \omega_{B∥}},$$

and

$$E_{0}=-\frac{\omega_{B⊥}^{2}/2}{f_{c}-\omega_{B∥}}-\frac{\omega_{B⊥}^{2}/2}{f_{c}+\omega_{B∥}},$$

where *ω_B_*_‖_ is the Zeeman energy owing to the longitudinal magnetic field, *ω_B_*_⊥_ is the Zeeman energy owing to the transverse magnetic field and *f_c_* = *D*(*T*) is the temperature-dependent zero-field splitting. Hence, the resonance frequencies *R*_L_ and *R*_H_ reflect the four magnetic parameters (*B*_0_, *θ*, *αI*, *φ*) as

4.1 $$\frac{R_{\text{H}}+R_{\text{L}}}{2}=f_{c}+\frac{3f_{c}\omega_{B⊥}^{2}}{2(f_{c}^{2}-\omega_{B∥}^{2})},$$

4.2 $$\frac{R_{\text{H}}-R_{\text{L}}}{2}=\omega_{B∥}-\frac{\omega_{B⊥}^{2}\omega_{B∥}}{2(f_{c}^{2}-\omega_{B∥}^{2})},$$

$$f_{c}=D(T),$$

$$\omega_{B∥}=\gamma (B_{0}\text{cos}\theta +\alpha I\text{cos}\varphi ),$$

$$\;\;\omega_{B⊥}=\gamma (B_{0}\text{sin}\theta +\alpha I\text{sin}\varphi ).$$

The theoretical value of the relative resonance frequency midpoint ΔRFM obtained using equation (4.1) is superimposed on the experimental value obtained in figure 8*b*. Although the time is shown as the horizontal axis in figure 8*b*, the busbar current is shown as the horizontal axis in figure 8*c*. In figure 8*c*, the experimental values for the first half increase from 0 to 340 A were plotted to eliminate the heating effect.

The values of *θ* and *φ* were set to minimize the deviation between the theoretical and experimental values. These angles *θ* and *φ* were not added intentionally but could have originated from imperfect parallelism between the sensor head and the busbar surface, as well as the non-uniform adhesive thickness between the fibre top and the diamond sensor surface.

ΔRFM also reflects the temperature change. The experimental value was lower than the theoretical value when changing from 340 to 0 A, as shown in figure 8*b*. This is considered to have resulted from the heat generated by the busbar current of the triangular wave, transferred to the diamond sensor with a delay owing to the thermal capacitance of the busbar and the surrounding sensor holder.

Notably, *θ* and *φ* also slightly influence the RFD. The term other than the Zeeman effect, the second term in (2) multiplied by 2, is shown in figure 8*c* as Δ_⊥_(RFD_B − RFD_A), as a purple dotted line. It was less than 0.1 MHz for a 340 A current, which corresponds to 0.01% of the RFD, Δ(RFD_B − RFD_A), and did not affect the normalized gain of the diamond sensor as shown in figure 8*d*.

In summary, the effect of the transverse magnetic field was observed as a change in the resonance frequency midpoint. The transverse magnetic field could have originated from the misalignment of the NV-axis to the magnetic field. For a several-degree misalignment and under a 340 A current, the effect of the transverse magnetic field on the linearity was negligibly small.

## 5. Summary

As an EV battery monitor with diamond quantum sensor, the realization of a dynamic range of ±1000 A with an accuracy of 10 mA, operation from −40 to 85°C, and SOC measurement accuracy of less than 0.2% in the standard WLTC current pattern, had already been reported based on the compact sensor head including a fibre-top diamond sensor that could be attached on a busbar, differential measurement and integral feedback circuit for accurate charge tracking.

To demonstrate the application capability of the EV battery monitor with a diamond quantum sensor, we investigated:

(i) Measurable current in a real car noise level.
(ii) Linearity comparison with conventional sensors.

For (i) and (ii), we further introduced a Helmholtz coil to which a variable external magnetic field could be applied in addition to the static magnetic field, as well as a Hall sensor and a shunt resistor connected in series to the busbar, which can simultaneously measure the busbar current with the diamond sensor.

As a result,

(i) A 20 mA current pulse could be measured even under an external magnetic field noise of 80 µT, which is larger than that of 50 µT around the EV battery module during driving in a real car.
(ii) The linearity degradation seen in the Hall sensor near the upper limit of the measurement range was not seen in the diamond sensor. Although the Hall sensor and the shunt resistor measurement errors increased in the small current range of several tens of amperes or less, the increase was smaller for the diamond sensor.

In the measurement of (ii), the effect of the transverse magnetic field was observed as a change in the resonance frequency midpoint. The transverse magnetic field could have originated from imperfect parallelism between the sensor head and the busbar surface, as well as the non-uniform adhesive thickness between the fibre top and the diamond sensor surface. The effect of the transverse magnetic field on the RFD was estimated to be 0.01% for a several-degree misalignment and under a 340 A current, and the effect on the linearity was negligibly small.

The measurable current was 10 mA and the SOC estimation accuracy was 0.1% in an unshielded environment but without intentional external noise. The SOC estimation accuracy degradation was predicted to be approximately 0.2% even when the measurable current became 20 mA owing to in-vehicle noise. The improvement from the current estimation accuracy of 10–0.1% or 0.2% makes no significant difference and has no impact on the CO_2_ reduction effect.

To further improve the measurability from 20 mA in (i), the distance between the two differential sensors should be decreased to improve the common-mode rejection ratio. For detailed discussions of linearity in (ii), improvement of the accuracy of the current source and the evaluation of multiple sensors of the same type would be necessary.

## Data Availability

The data that support the findings of this study are available from the corresponding author upon reasonable request.
